# Wife-husband differences in the associations between sleep durations and global cognition among middle-aged and older married couples in China: a longitudinal dyadic analysis

**DOI:** 10.1093/gerona/glag235

**Published:** 2026-09-17

**Authors:** Jie Zhong, Ying Dai, Xiang Qi, Wei Liang

**Affiliations:** School of Nursing, Li Ka Shing Faculty of Medicine, The University of Hong Kong, Hong Kong, China; School of Nursing, Li Ka Shing Faculty of Medicine, The University of Hong Kong, Hong Kong, China; Rory Meyers College of Nursing, New York University, New York, New York, United States; School of Nursing, Nanjing Medical University, Nanjing, China

**Keywords:** Sleep duration, Cognitive function, Dyadic analysis, Actor-Partner Interdependence Model, CHARLS

## Abstract

**Background:**

Sleep duration has well-established U-shaped associations with cognitive health, but research has largely ignored spousal interdependence. This study examined actor and partner effects of sleep duration on global cognition in middle-aged and older Chinese couples, testing whether associations differed by sex.

**Methods:**

Data were drawn from CHARLS waves 1-4 (2011-2018). The analytical sample comprised 4260 couples (8520 individuals) aged ≥45 years living in a married, 2-person, mixed-sex household. Global cognition was assessed with a composite battery adapted from the Telephone Interview for Cognitive Status and Chinese Mini-Mental State Examination; nocturnal sleep and napping durations were self-reported. Actor-Partner Interdependence Models with quadratic terms examined actor effects (own sleep predicting own cognition) and partner effects (own sleep predicting spouse’s cognition), with false discovery rate (FDR)-adjusted *q*-values and sex-equality tests.

**Results:**

Significant inverted U-shaped actor effects were found for wives (*β* = −0.010, 95% CI: −0.014, −0.005, *p* < .001) and husbands (*β* = −0.011, 95% CI: −0.016, −0.006, *p* < .001); both survived FDR correction (*q* = 0.002, *q* < 0.001). Husbands’ sleep was nominally associated with wives’ cognition similarly (*β* = −0.005, *p* = .025), while wives’ sleep was not associated with husbands’ cognition (*p* = .618). The husband-to-wife association did not survive FDR correction (*q* = 0.239), and sex-equality tests were non-significant (*χ*²(9) = 9.35, *p* = .406). Napping showed limited, largely non-significant associations with cognition.

**Conclusions:**

Own nocturnal sleep duration shows a robust, symmetric inverted U-shaped association with cognitive function in both spouses. The possible husband-to-wife partner effect did not survive correction for multiple testing and warrants adequately powered investigation before informing couple-based clinical recommendations.

## Introduction

Alzheimer’s disease and related dementia is a public health problem across the world. The number of people with dementia is about 50 million globally and will increase to 152 million by 2050.^1^ In China, there are more than 10 million people with dementia, accounting for 25% of the patients with dementia in the world.^2^ Cognitive impairment is the cardinal feature during the long preclinical period of dementia, which significantly increases the risk of functional dependence and poor quality of life in older adults.^3^ The global cost of dementia is estimated to exceed US $1.3 trillion, with informal caregiving accounting for approximately half of this expenditure.^1^ Therefore, identifying and modifying risk factors for the prevention and delay of cognitive impairment is clinically important among older adults, family caregivers, and health and social care systems,^4^ with emerging and growing interest in the role of sleep behaviors, such as the optimal duration of nighttime sleep and napping.

Sleep problems have long been associated with cognition and risk of cognitive impairment or dementia. Epidemiological studies indicated that up to 90% of patients experience disrupted sleep before the emergence of cognitive impairment.^5^^,^^6^ A meta-analysis estimated that about 15% of Alzheimer’s disease in the population may be due to sleep problems.^7^ Several dose-response meta-analyses identified a non-linear, U-shaped association between total sleep duration and the risk of cognitive decline.^8–11^ Still, evidence on the U-shaped association between sleep duration and cognitive health remains inconclusive. A study in China using longitudinal data observed a U-shaped association between nocturnal sleep and risk of cognitive impairment,^12^ whereas a Swedish study specifically linked decreased sleep duration with dementia incidence^13^ and a large prospective cohort study in the United States showed that elevated risk of cognitive decline was observed among women reporting shorter sleep.^14^ Additionally, the relationship between sleep duration and cognition has often been studied at a single time point or immediately following follow-up,^8^^,^^9^ while ignoring the dynamic nature of sleep and its duration throughout the life course. Therefore, further exploration into the associations between shorter and longer sleep durations and cognitive function across time points is essential.

Habitual afternoon napping, such as routinely taking a nap after lunch, is prevalent in Chinese older adults and is considered part of a healthy lifestyle from a cultural perspective.^15^^,^^16^ The post-lunch circadian dip period from 1:00 to 4:00 pm is the most common and preferred time for naps. Some but limited studies reported the short-term benefits of moderate nap duration (eg, 10 min) on cognition such as greater alertness, accuracy, and speed when performing cognitive tasks,^17^^,^^18^ while longer naps (eg, >30 min) might lead to a transient deterioration in cognitive performance.^18^ One study has supported a similar inverted U-shaped association between napping and cognition, suggesting that absence of napping and too much napping were associated with worse cognition, but naps with a moderate duration were associated with better cognition and may be an important part of optimizing cognition in older adults.^19^ However, more rigorous evidence is needed to clarify whether no napping or extended napping predicts cognitive change.

Although sleep durations and cognition have been linked at the individual level, the spouse’s role in later life remains understudied. For many older adults, a spouse is the most significant social partner, and decades of life course research underscore the “linked lives” in which health and well-being are profoundly interdependent between partners.^20^ Marriage is a relationship wherein the behaviors, perceptions, and health of 1 spouse often have consequences for the other. Consistent with interdependence theory, a large body of evidence supports that individuals are more likely to lose weight, quit smoking, and stay physically active if their spouses make the positive change.^21^ Much like sleep problems and cognitive impairment may be shared through partners’ needs for and provision of support, as well as their shared daily activities and resources.^22^ Specifically, cognitive trajectories of aging couples are correlated, and close partners have the ability to prompt and facilitate memory performance in both healthy aging and Alzheimer’s disease progression.^23^

Methodologically, longitudinal dyadic analysis using the Actor and Partner Interdependence Model (APIM) has been applied in psychology, family science, and gerontology in the past 2 decades, but mostly in Western cultures.^24^ In older age, burdens faced by couples dealing with health problems are inordinately shouldered by women, with wives experiencing greater detriments to their wellbeing from their husbands’ health problems than husbands experience from wives’ health problems.^22^ Some evidence has established that 1 partner’s status (eg, social engagement, marital satisfaction) has dyadic effects for both partners’ wellbeing (eg, depression, cognitive impairment) among older married couples, with sex differences between wives and husbands.^22^^,^^25^^,^^26^ Applying longitudinal APIM in Chinese married middle-aged and older couples, this study aimed to establish the inverted U-shaped associations between each partner’s sleep duration and cognitive function and to identify the direction and magnitude of crossover effects between wives and husbands. Research questions included (1) whether one’s own nocturnal sleep and post-lunch napping durations were associated with concurrent cognitive function, (2) whether 1 spouse’s sleep durations were associated with the other spouse’s cognitive function concurrently, and (3) whether actor and partner effects differed between wives and husbands.

## Methods

### Study Population

This study used data from the China Health and Retirement Longitudinal Study (CHARLS), a nationally representative longitudinal survey of middle-aged and older adults in China. The baseline survey was conducted in 2011, with follow-up waves in 2013, 2015, 2018, and 2020. Wave 5 (2020) was excluded from the primary analysis due to COVID-19-related disruptions in sleep patterns and cognitive assessment.

The inclusion criteria for couples were (1) married, (2) living in the same household, (3) 1 woman and 1 man, (4) both partners aged 45 years and older. Couples were retained in the dyadic panel only while both spouses continued to be observed as a married, 2-person, mixed-sex household at each successive wave. When this household structure no longer held, the couple was censored from the analysis from that wave onward. Couples were required to have complete nocturnal sleep duration and global cognition data at baseline for both spouses to enter the analytic sample; no other variable, including covariates, was required to be complete for cohort entry. Couples were not required to have complete 4-wave follow-up to be included. After applying these criteria, the baseline analytic sample comprised 4260 couples. In our longitudinal models, wave was coded as time elapsed since baseline (0, 2, 4, and 7 years for Waves 1-4, respectively) to account for the unequal spacing between waves. Of the *N* = 4260 couples in the baseline analytic sample, 3496 (82.1%) contributed data at Wave 2, 2959 (69.5%) at Wave 3, and 2445 (57.4%) at Wave 4.

### Measures

#### Sleep durations

Sleep durations included nocturnal sleep and post-lunch napping. Nocturnal sleep duration was determined by a self-report item: “During the past month, how many hours of actual sleep did you get at night? (This may be shorter than the number of hours you spend in bed.)” Responses were recorded in hours and treated as a continuous variable in all analyses. Post-lunch napping duration was assessed by asking, “During the past month, how long did you take a nap after lunch in general?” Responses were recorded in minutes and likewise treated as a continuous variable in all analyses.

#### Global cognition

Cognitive function was assessed using a composite battery adapted from items used in the Telephone Interview for Cognitive Status (TICS) and the Chinese version of the Mini-Mental State Examination (MMSE), which have been applied in previous studies.^27^^,^^28^ The cognitive assessment comprised 4 domains: orientation, memory, executive function, and calculation, following standard CHARLS protocols. Each domain was scored by counting the number of individually correct responses within that domain (orientation: 0-5; immediate recall: 0-10; delayed recall: 0-10; figure copying: 0-1; serial-7s: 0-5), and the 4 domain scores were summed to produce a single composite ranging from 0 to 31, with higher scores indicating better cognitive function.

*Orientation* was assessed using 5 questions measuring awareness of time and place. Respondents were asked to report the current year, month, day of the month, day of the week, and season. Each correct response was scored as 1 point, with incorrect or missing responses scored as 0. Higher scores indicate better orientation. *Memory* was assessed using both immediate and delayed word recall tasks. First, interviewers read a list of 10 common Chinese nouns at a rate of approximately 1 word/s. Respondents were instructed to recall as many words as possible immediately after hearing the list. The total number of correctly recalled words was recorded, with each correct word contributing 1 point. Approximately 5 min after the immediate recall task (during which time other cognitive tests were administered), respondents were asked to recall the same 10-word list. The total number of correctly recalled words was recorded, with each correct word contributing 1 point. The delayed recall score reflects episodic memory and consolidation ability. Both immediate and delayed recall scores range from 0 to 10. *Executive function* was assessed using a figure drawing task. Respondents were asked to copy 2 intersecting pentagons, a task that requires visuospatial constructional ability and executive function. The drawing was scored as 1 point if the figure was correctly reproduced (ie, 2 pentagons intersecting to form a 4-sided figure) and 0 points if incorrectly drawn or missing. *Calculation ability* was assessed using the serial 7s subtraction task, a standard measure of attention and working memory. Respondents were asked to subtract 7 from 100, and then repeatedly subtract 7 from the remainder for a total of 5 trials (ie, 93, 86, 79, 72, 65). Each correct subtraction was scored as 1 point, yielding a total score ranging from 0 to 5. The task was discontinued if the respondent made an error, but subsequent correct responses were not counted, following standard scoring protocols.

#### Covariates

Both partners’ covariates were entered simultaneously into each equation to account for mutual confounding, except for menopausal status for wives.

##### Demographics

Age was calculated based on self-reported birth year and survey year. Educational level was categorized based on the Chinese education system: illiterate, elementary school, middle school, and high school or above. Current hukou status was classified as agricultural or nonagricultural. Work status was coded as retired or not retired.

##### Menopausal status

Wives were asked whether they had started menopause (yes/no). This item was included as a covariate in wives’ models only.

##### Functional status

ADL (Activities of Daily Living) disability was assessed using 6 items from the Katz Index of Independence in ADL: dressing, bathing, eating, getting in and out of bed, using the toilet, and controlling urination and defecation. ADL disability was defined as having difficulty with at least 1 activity or no difficulty with any activity. IADL (Instrumental Activities of Daily Living) disability was assessed using 5 items from the Lawton IADL Scale: household chores, cooking, shopping, managing money, and taking medications. IADL disability was defined as having difficulty with at least 1 activity or no difficulty with any activity.

##### Multimorbidity

Participants were asked whether they had ever been diagnosed by a physician with any of 14 chronic conditions: hypertension, dyslipidemia, diabetes, cancer, chronic lung disease, liver disease, heart disease, stroke, kidney disease, digestive disease, emotional/psychiatric problems, memory-related disease, arthritis/rheumatism, and asthma. Multimorbidity was defined as the presence of 2 or more chronic conditions.

##### Falls in the past 2 years

History of falls was assessed by asking participants whether they had fallen in the past 2 years and coded as yes (had a fall) or no (did not have a fall).

##### Depression

Depressive symptoms were assessed using the 10-item Center for Epidemiologic Studies Depression Scale (CES-D-10). Respondents rated the frequency of 10 symptoms during the past week on a 4-point scale: 0 = rarely (<1 day), 1 = some days (1-2 days), 2 = occasionally (3-4 days), 3 = most days (5-7 days). The total score ranged from 0 to 30, with higher scores indicating more depressive symptoms. Following validated cutoffs for Chinese older adults,^29^ clinically significant depressive symptoms were defined as a score of 12 or higher in older populations.

##### Risk behaviors

Smoking status was categorized as current smokers and nonsmokers (eg, never smokers or ex-smokers). Drinking status was categorized as heavy drinkers and non-heavy drinkers, including nondrinkers. Heavy drinking followed guideline thresholds: >14 drinks/week for men <65 years, >7 drinks/week for women <65 years, and >7 drinks/week for all adults ≥65 years.^30^

##### Bodily pain

Participants were asked whether they currently experienced any bodily pain (yes/no).

##### Social participation

Social participation was assessed using a series of questions about engagement in 11 social activities during the past month. Based on prior CHARLS studies,^27^^,^^31^ we selected 7 activities as social participation: “interacting with friends,” “playing mahjong, cards, or chess,” “going to a sport or social club,” “participating in a community organization,” “volunteering or charity work,” “caring for a sick or disabled adult living apart,” and “attending an educational or training course.” Social inactivity was defined as a score of 0 (no participation in any activity).

##### Body mass index

Anthropometric measurements, including height, weight, and waist circumference, were collected during physical examinations conducted by trained researchers following standardized protocols. Body mass index (BMI) was calculated as weight in kilograms divided by height in meters squared (kg/m²).

##### Physical activity levels

Physical activity was assessed using the International Physical Activity Questionnaire (IPAQ) short form. Participants reported weekly frequency and categorical duration (<30 min, 30 min to 2 h, >2 h) for vigorous, moderate, and walking activities. Midpoint durations (15, 75, and 150 min) were assigned to calculate weekly minutes. MET-min/week were computed as (vigorous minutes × 8) + (moderate minutes × 4) + (walking minutes × 3.3).

### Statistical Analysis

All statistical analyses were performed using R (version 4.x). A 2-sided *p*-value <.05 was considered statistically significant. Baseline characteristics of wives and husbands were described separately using means and standard deviations (*SD*) for continuous variables and frequencies with percentages for categorical variables. Differences between wives and husbands were examined using paired *t*-tests for continuous variables and McNemar’s tests for categorical variables, accounting for the paired nature of the data. Couples were required to have non-missing nocturnal sleep duration and cognition score at baseline (Wave 1) for both spouses to enter the analytic sample (*N* = 4260). No other variable was required to be complete for cohort entry, and adjusted models used listwise deletion for any covariate with missing values, with no data imputation.

To examine the longitudinal, bidirectional associations between sleep duration and the risk of cognitive impairment over time within couples, we employed the APIM for distinguishable dyads (husbands and wives). This approach accounts for the statistical interdependence within couples and simultaneously estimates 2 types of effects: actor effects, which represent the association between an individual’s own sleep duration and their own cognition measured concurrently at the same wave, and partner (crossover) effects, which represent the association between 1 partner’s sleep duration and the other partner’s concurrently measured cognition. Sleep duration and cognition were both time-varying, assessed at every wave (Waves 1-4, coded as 0, 2, 4, and 7), and the Actor × Time and Partner × Time interaction terms tested whether the shape of these concurrent associations changed across waves.

Given the well-established U-shaped relationship between sleep duration and cognitive outcomes,^9^^,^^10^ we specified non-linear APIMs by including linear and quadratic terms for each sleep measure. The model was specified as follows. For wife *i* in couple *j*, cognition was modeled as a function of her own concurrent linear and quadratic sleep-duration terms, her husband’s concurrent corresponding sleep-duration terms, time, the interactions of time with each of these terms, and covariates from both partners. For husband *i* in couple *j*, cognition was modeled symmetrically as a function of his own concurrent linear and quadratic sleep-duration terms, his wife’s concurrent corresponding sleep-duration terms, time, the interactions of time with each of these terms, and covariates from both partners. In these equations, the coefficients for wives’ and husbands’ sleep-duration terms represent actor effects (own sleep predicting own cognition), while the coefficients for the spouse’s variables represent partner effects (own sleep predicting the partner’s cognition).

Multilevel modeling was used to estimate APIM. Models included 2 levels: level 1 represents within-person variability, and level 2 represents between-couple variability. Models were estimated using multilevel modeling with maximum likelihood estimation. Continuous variables were grand-mean-centered prior to analysis. The raw composite score was standardized separately within each wave: for each wave, we computed the wave-specific sample mean and standard deviation of the raw composite among all respondents assessed in that wave, and converted each individual’s raw score to a z-score as (raw score − wave mean)/wave *SD*. The residuals of wives’ and husbands’ outcomes were allowed to covary to account for unobserved shared couple-level factors, and robust standard errors were used to account for non-independence within couples. The effect of time was tested in each model, and the intercept and slope of time were allowed to vary within and between couples. Following recommended procedures,^26^ sex (1 = wife, −1 = husband) was used as a distinguishing variable to examine separate intercepts and slopes for wives and husbands. To ensure the parameter to identify the deviation of a variable from the grand mean of the outcome, continuous predictors were grand-mean centered. Model fit for each APIM was assessed using Akaike Information Criterion (AIC), Bayesian Information Criterion (BIC), and log-likelihood. Additionally, turning points (the sleep duration associated with maximal predicted cognition) were estimated from the quadratic term coefficients, with 95% confidence intervals derived via the delta method. We also fit restricted cubic spline models for significant results as a flexible alternative to the quadratic specification that does not assume a symmetric curve.

#### Multiple testing

To formally test whether the sleep-cognition association differed by sex, we fit a pooled model interacting every actor, partner, and time term with partner sex, and compared it against the same model without these interactions via a likelihood-ratio test. We also tested the actor and partner effects for sex-equality directly using joint Wald tests. Given the number of actor, partner, and time-interaction effects tested across the nocturnal sleep, napping, and total sleep models, we applied the Benjamini-Hochberg procedure to control the false discovery rate across this family of 32 tests including the nocturnal sleep duration and napping duration models, and report (false discovery rate) FDR-adjusted *q*-values alongside nominal *p*-values.

#### Sensitivity analyses

(2) *Standardization approach.* We re-estimated the fully adjusted models using the raw (unstandardized) cognition score and a Wave-1-baseline-referenced standardization, in addition to our primary wave-standardized outcome, to confirm our conclusions did not depend on the standardization approach. (2) *Expanded covariate set.* A sensitivity analysis using an expanded covariate set (additionally including BMI, MET-min physical activity, ADL, IADL, and self-rated health).

## Results

Table 1 presents the baseline characteristics of the final analytic sample of 4260 couples. Compared with wives, husbands were older (59.38 vs 57.29 years, *p* < .001), more likely to have higher educational attainment, and more likely to smoke and drink heavily (all *p* < .001). Wives had higher levels of IADL difficulty, falls, bodily pain, and depressive symptoms than husbands (all *p* < .001); ADL difficulty did not differ significantly between spouses (*p* = .591). Husbands reported longer nocturnal sleep (6.51 vs 6.31 h, *p* < .001), longer post-lunch napping (37.38 vs 26.93 min, *p* < .001), and higher cognitive scores (15.32 vs 13.80, *p* < .001) than wives. Among wives, 72.42% were postmenopausal at baseline. Baseline couple-level (wife-husband) correlations were *r* = 0.233 for nocturnal sleep duration, *r* = 0.320 for napping duration, and *r* = 0.453 for cognition score (all *p* < .001).

**Table 1:** Baseline characteristics of wives and husbands (*N* = 4260 couples).

Study variables	Wives (*N* = 4260)	Husbands (*N* = 4260)	*p* value
**Age (years)**	57.29 ± 8.39	59.38 ± 8.96	**<.001**
45 to <55	1723 (40.45%)	1383 (32.46%)	
55 to <65	1735 (40.73%)	1755 (41.20%)	
65 to <75	656 (15.40%)	841 (19.74%)	
75+	146 (3.43%)	281 (6.60%)	
**Education level**			**<.001**
Illiterate	1451 (34.06%)	416 (9.77%)	
Elementary school	1559 (36.60%)	1819 (42.70%)	
Middle school	799 (18.76%)	1213 (28.47%)	
High school+	450 (10.56%)	812 (19.06%)	
Missing	1 (0.02%)	0 (0.00%)	
**Current hukou status**			**<.001**
Agricultural	3259 (76.50%)	3056 (71.74%)	
Nonagricultural	1001 (23.50%)	1204 (28.26%)	
**Work status**			**<.001**
Not retired	3671 (86.17%)	3523 (82.70%)	
Retired	540 (12.68%)	729 (17.11%)	
Missing	49 (1.15%)	8 (0.19%)	
**Menopausal status (wives only)**			
Postmenopausal	3085 (72.42%)	N/A	
Premenopausal	1151 (27.02%)	N/A	
Missing	24 (0.56%)	N/A	
**Smoking status**			**<.001**
Nonsmokers	4011 (94.15%)	1874 (43.99%)	
Current smokers	249 (5.85%)	2386 (56.01%)	
**Heavy drinking**			**<.001**
Non-heavy drinkers	4242 (99.58%)	3735 (87.68%)	
Heavy drinkers	18 (0.42%)	524 (12.30%)	
Missing	0 (0.00%)	1 (0.02%)	
**Social participation**			**<.001**
Socially active	1546 (36.29%)	1408 (33.05%)	
Socially inactive	2714 (63.71%)	2852 (66.95%)	
**ADL**			.591
No difficulty	3078 (72.25%)	2676 (62.82%)	
Any difficulty	717 (16.83%)	631 (14.81%)	
Missing	465 (10.92%)	953 (22.37%)	
**IADL**			**<.001**
No difficulty	3395 (79.69%)	3651 (85.70%)	
Any difficulty	864 (20.28%)	606 (14.23%)	
Missing	1 (0.02%)	3 (0.07%)	
**Self-rated health**			**<.001**
Good/fair	2523 (59.23%)	2802 (65.77%)	
Poor	1139 (26.74%)	860 (20.19%)	
Missing	598 (14.04%)	598 (14.04%)	
**Fell in the past 2 years**			**<.001**
No fall	3567 (83.73%)	3697 (86.78%)	
Had a fall	692 (16.24%)	562 (13.19%)	
Missing	1 (0.02%)	1 (0.02%)	
**Bodily pain**			**<.001**
No pain	2751 (64.58%)	3267 (76.69%)	
Any pain	1508 (35.40%)	993 (23.31%)	
Missing	1 (0.02%)	0 (0.00%)	
**Depression (CES-D** ≥**12)**			**<.001**
No depression	2600 (61.03%)	3122 (73.29%)	
Depression	1635 (38.38%)	1112 (26.10%)	
Missing	25 (0.59%)	26 (0.61%)	
**Multimorbidity**			**.00436**
None	1361 (31.95%)	1441 (33.83%)	
Single	1176 (27.61%)	1253 (29.41%)	
Multimorbidity (≥2)	1583 (37.16%)	1452 (34.08%)	
Missing	140 (3.29%)	114 (2.68%)	
**BMI (kg/m^2^)**	24.35 ± 3.98	23.35 ± 3.73	**<.001**
**Physical activity (MET-min/week)**	4831.82 ± 4445.12	5408.74 ± 4803.41	**<.001**
**Nocturnal sleep (h/night)**	6.31 ± 1.80	6.51 ± 1.72	**<.001**
**Post-lunch napping (min/day)**	26.93 ± 39.27	37.38 ± 43.13	**<.001**
**Global cognition score**	13.80 ± 5.95	15.32 ± 5.29	**<.001**

ADL/IADL = activities/instrumental activities of daily living (any difficulty in ≥1 item); CES-D = depression scale (0-30); BMI = kg/m²; MET = physical activity, MET-min/week; heavy drinking followed guideline thresholds: >14 drinks/week for men <65 years, >7 drinks/week for women <65 years, and >7 drinks/week for all adults ≥65 years; socially active = ≥1 social activity/month; self-rated health poor = “poor”/“very poor” of 5 levels; cognition score = 0-31 composite (orientation, recall, figure copying, serial-7s); menopausal status assessed in wives only. Wife-husband comparisons: paired *t*-tests (continuous); McNemar’s test (binary categorical); Bowker’s test of symmetry (multi-level categorical: age band, education, multimorbidity). Bold values indicate significant differences by p values < 0.05.

Complete-case sample sizes for every model are reported in Table S1. Random-effects variance components and model fit statistics (AIC, BIC, log-likelihood) for each primary adjusted model are described in Table S2. Full nominal and FDR-adjusted *q*-values for all 32 actor, partner, and time-interaction tests across the nocturnal sleep and napping models are reported in Table S3.

### Nocturnal Sleep Duration and Global Cognition

Table 2 presents the APIMs examining the associations between nocturnal sleep duration and global cognition score.

**Table 2:** Dyadic model of actor and partner effects of nocturnal sleep duration on global cognition.

	Parameters	Unadjusted					Adjusted				
		*β*	*SE*	95% CI lower	95% CI upper	*p* value	*β*	*SE*	95% CI lower	95% CI upper	*p* value
Wives	Intercept	0.0804	0.0176	0.0459	0.1149	<.001	0.3548	0.1606	0.0401	0.6696	.027
	Time	−0.0412	0.0049	−0.0508	−0.0315	<.001	−0.0345	0.0048	−0.0440	−0.0250	<.001
	Actor (own sleep)—linear	0.0182	0.0068	0.0049	0.0315	.007	−0.0026	0.0065	−0.0153	0.0101	.687
	Actor (own sleep)—quadratic	−0.0185	0.0025	−0.0234	−0.0136	<.001	−0.0095	0.0024	−0.0142	−0.0048	<.001
	Partner (spouse’s sleep)—linear	0.0067	0.0071	−0.0071	0.0206	.339	−0.0012	0.0068	−0.0144	0.0121	.864
	Partner (spouse’s sleep)—quadratic	−0.0133	0.0024	−0.0181	−0.0086	<.001	−0.0053	0.0024	−0.0099	−0.0007	.025
	Actor × Time—linear	−0.0022	0.0022	−0.0064	0.0020	.305	0.0002	0.0021	−0.0039	0.0044	.917
	Actor × Time—quadratic	4.96e − 5	0.0008	−0.0016	0.0017	.952	−0.0013	0.0008	−0.0028	0.0003	.116
	Partner × Time—linear	−0.0005	0.0022	−0.0047	0.0038	.834	0.0018	0.0022	−0.0024	0.0060	.405
	Partner × Time—quadratic	0.0026	0.0007	0.0012	0.0041	<.001	0.0015	0.0007	0.0001	0.0029	.034
Husbands	Intercept	0.0866	0.0179	0.0515	0.1216	<.001	0.6563	0.1628	0.3372	0.9755	<.001
	Time	−0.0227	0.0049	−0.0322	−0.0131	<.001	−0.0193	0.0049	−0.0289	−0.0097	<.001
	Actor (own sleep)—linear	0.0047	0.0075	−0.0100	0.0193	.532	−0.0146	0.0073	−0.0288	−0.0003	.045
	Actor (own sleep)—quadratic	−0.0228	0.0026	−0.0279	−0.0177	<.001	−0.0115	0.0026	−0.0165	−0.0064	<.001
	Partner (spouse’s sleep)—linear	0.0139	0.0071	2.34e − 5	0.0277	.050	0.0048	0.0069	−0.0087	0.0183	.483
	Partner (spouse’s sleep)—quadratic	−0.0071	0.0026	−0.0122	−0.0020	.006	−0.0013	0.0025	−0.0062	0.0037	.618
	Actor × Time—linear	−0.0033	0.0022	−0.0076	0.0009	.124	−0.0012	0.0022	−0.0054	0.0031	.593
	Actor × Time—quadratic	0.0006	0.0007	−0.0009	0.0020	.445	−0.0007	0.0007	−0.0022	0.0007	.333
	Partner × Time—linear	−0.0029	0.0020	−0.0069	0.0011	.155	−0.0020	0.0020	−0.0060	0.0021	.339
	Partner × Time—quadratic	0.0005	0.0007	−0.0010	0.0019	.529	−0.0001	0.0008	−0.0015	0.0014	.937

β = unstandardized regression coefficient; SE = standard error; CI = confidence interval.* N* = 4260 couples at baseline; complete-case *N* per model in Table S1 (adjusted models retained 92.4%-92.9%; listwise deletion, no imputation). Actor = own sleep → own cognition; partner = partner’s sleep → own cognition; Time = wave (centered at baseline). *β*/*SE* from linear mixed-effects models; *p*-values from Wald *t*-tests (Satterthwaite). Adjusted for age, education, current hukou status, smoking, heavy drinking, falls, bodily pain, CES-D, retirement, multimorbidity, and social participation; wives’ models add menopausal status.

#### Actor Effects (Own Sleep → Own Cognition)

For wives, the quadratic term was significant in both unadjusted (*β* = −0.0185, 95% CI: −0.0234, −0.0136, *p* < .001) and adjusted models (*β* = −0.0095, 95% CI: −0.0142, −0.0048, *p* < .001; FDR *q* = 0.002). For husbands, the quadratic term was significant in both unadjusted (*β* = −0.0228, 95% CI: −0.0279, −0.0177, *p* < .001) and adjusted models (*β* = −0.0115, 95% CI: −0.0165, −0.0064, *p* < .001; FDR *q* < 0.001).

Using the quadratic models, the estimated nocturnal sleep duration associated with optimal global cognition was 6.09 h (95% CI: 5.43, 6.76) for wives and 5.85 h (95% CI: 5.20, 6.50) for husbands. Predicted cognition scores across representative sleep durations are shown in Table S4, and the full predicted curve with 95% confidence bands, overlaid on the observed distribution of sleep duration, is shown in Figure 1. Furthermore, we fit restricted cubic splines (5 knots, Harrell percentile placement) for the actor’s own sleep-cognition relationship. The likelihood-ratio test confirmed significant nonlinearity beyond a linear term for both wives (*χ*²(3) = 18.13, *p* < .001) and husbands (*χ*²(3) = 22.47, *p* < .001). Full knot placement, nonlinearity test statistics, and spline-versus-quadratic predicted values are reported in Table S5.

**Figure 1:**
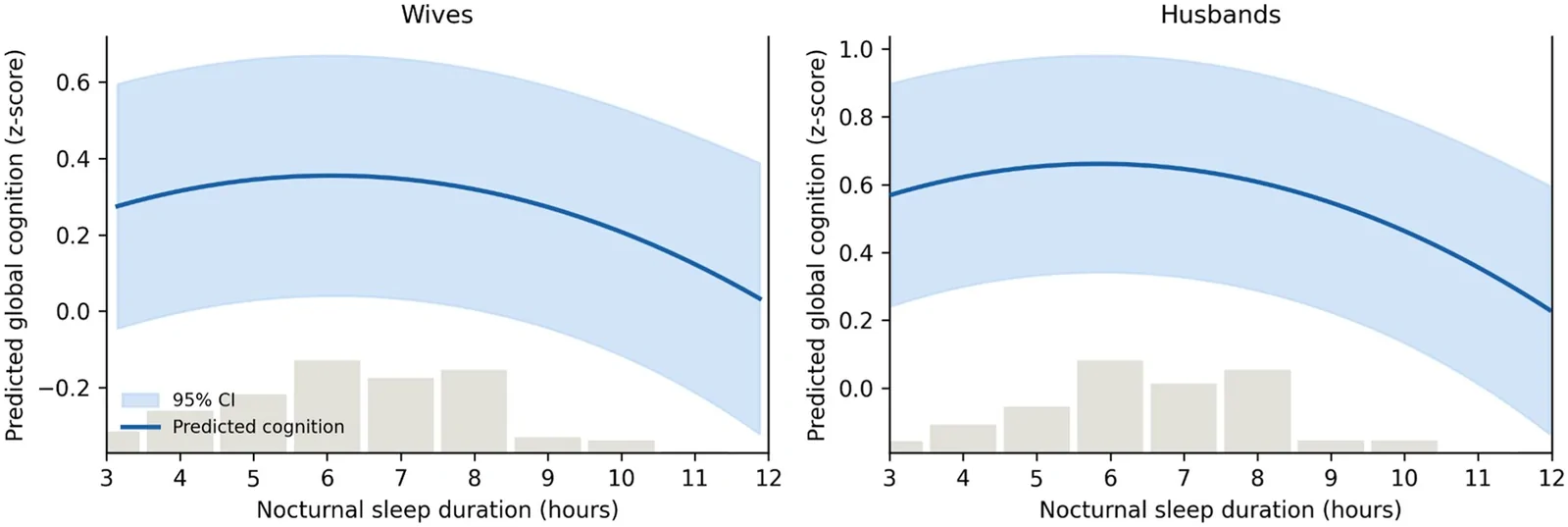
Predicted global cognition by nocturnal sleep duration, with 95% confidence bands and sample distribution (adjusted models).

#### Partner Effects (Spouse’s Sleep → Own Cognition)

Husbands’ sleep duration was nominally associated with wives’ global cognition in an inverted U-shaped pattern in unadjusted (*β* = −0.0133, 95% CI: −0.0181, −0.0086, *p* < .001) and adjusted models (*β* = −0.0053, 95% CI: −0.0099, −0.0007, *p* = .025; FDR *q* = 0.239), though this did not survive FDR correction. Wives’ sleep duration showed a significant unadjusted association with husbands’ global cognition (*β* = −0.0071, *p* = .006) that was fully attenuated after adjustment (*β* = −0.0013, 95% CI: −0.0062, 0.0037, *p* = .618; FDR *q* = 0.847).

#### Sex Differentiation

For nocturnal sleep, the omnibus test was not significant (*χ*²(9) = 9.35, *p* = .406). Joint Wald tests likewise found no significant sex difference in the actor effects (*χ*²(2) = 0.09, *p* = .957) or the partner effects (*χ*²(2) = 2.54, *p* = .280).

### Post-Lunch Napping Duration and Global Cognition

Table 3 presents the results for post-lunch napping. Wives’ own napping duration showed a significant inverted U-shaped association with their own global cognition in the unadjusted model (quadratic *β* = −2.07 × 10⁻⁵, *p* = .001) that was attenuated to non-significance after adjustment (quadratic *β* = −1.05 × 10⁻⁵, 95% CI: −2.25 × 10⁻⁵, 0.15 × 10⁻⁵, *p* = .086; FDR *q* = 0.376). A partner effect for husbands’ napping predicting wives’ cognition was nominally non-significant after adjustment (linear, adjusted *β* = −7.89 × 10⁻⁴, 95% CI: −15.89 × 10⁻⁴, 0.13 × 10⁻⁴, *p* = .054; FDR *q* = 0.258). No significant actor effect was observed for husbands’ napping (adjusted *β* = 6.91 × 10⁻⁴, *p* = .112), and no significant partner effect was observed for wives’ napping predicting husbands’ cognition (adjusted *β* = 7.39 × 10⁻⁵, *p* = .873).

**Table 3:** Dyadic model of actor and partner effects of post-lunch napping duration on global cognition.

	Parameters	Unadjusted					Adjusted				
		*β*	*SE*	95% CI lower	95% CI upper	*p* value	*β*	*SE*	95% CI lower	95% CI upper	*p* value
Wives	Intercept	0.0216	0.0195	−0.0166	0.0599	.267	0.3680	0.1615	0.0514	0.6845	.023
	Time	−0.0399	0.0052	−0.0501	−0.0298	<.001	−0.0362	0.0051	−0.0462	−0.0261	<.001
	Actor (own sleep)—linear	0.0015	0.0005	0.0006	0.0024	<.001	0.0007	0.0004	−0.0002	0.0015	.122
	Actor (own sleep)—quadratic	−2.07e − 5	6.37e − 6	−3.31e − 5	−8.17e − 6	.001	−1.05e − 5	6.11e − 6	−2.25e − 5	1.49e − 6	.086
	Partner (spouse’s sleep)—linear	−0.0007	0.0004	−0.0015	0.0002	.119	−0.0008	0.0004	−0.0016	1.23e − 5	.054
	Partner (spouse’s sleep)—quadratic	6.98e − 7	5.48e − 6	−1.00e − 5	1.14e − 5	.899	7.58e − 6	5.31e − 6	−2.82e − 6	1.80e − 5	.153
	Actor × Time—linear	−0.0002	0.0001	−0.0005	0.0001	.240	1.54e − 5	0.0001	−0.0003	0.0003	.915
	Actor × Time—quadratic	1.32e − 6	2.00e − 6	−2.60e − 6	5.25e − 6	.508	−6.05e − 7	1.95e − 6	−4.43e − 6	3.22e − 6	.756
	Partner × Time—linear	0.0002	0.0001	−0.0001	0.0004	.223	0.0001	0.0001	−0.0001	0.0004	.282
	Partner × Time—quadratic	−4.38e − 7	1.52e − 6	−3.42e − 6	2.54e − 6	.774	−3.41e − 7	1.55e − 6	−3.38e − 6	2.70e − 6	.826
Husbands	Intercept	0.0422	0.0198	0.0034	0.0810	.033	0.6918	0.1637	0.3709	1.0126	<.001
	Time	−0.0267	0.0049	−0.0364	−0.0170	<.001	−0.0228	0.0049	−0.0325	−0.0132	<.001
	Actor (own sleep)—linear	0.0012	0.0005	0.0003	0.0021	.007	0.0007	0.0004	−0.0002	0.0015	.112
	Actor (own sleep)—quadratic	−1.72e − 5	5.78e − 6	−2.86e − 5	−5.91e − 6	.003	−7.25e − 6	5.63e − 6	−1.83e − 5	3.78e − 6	.198
	Partner (spouse’s sleep)—linear	0.0007	0.0005	−0.0003	0.0016	.160	0.0001	0.0005	−0.0008	0.0010	.873
	Partner (spouse’s sleep)—quadratic	−1.21e − 5	6.59e − 6	−2.50e − 5	8.12e − 7	.066	−4.92e − 6	6.44e − 6	−1.75e − 5	7.70e − 6	.445
	Actor × Time—linear	−0.0003	0.0001	−0.0005	−0.0001	.010	−0.0003	0.0001	−0.0005	−3.78e − 5	.023
	Actor × Time—quadratic	2.61e − 6	1.19e − 6	2.71e − 7	4.95e − 6	.029	1.23e − 6	1.18e − 6	−1.09e − 6	3.55e − 6	.300
	Partner × Time—linear	1.08e − 5	0.0001	−0.0003	0.0003	.938	0.0001	0.0001	−0.0001	0.0004	.335
	Partner × Time—quadratic	1.03e − 6	1.73e − 6	−2.36e − 6	4.41e − 6	.552	−1.74e − 7	1.72e − 6	−3.55e − 6	3.20e − 6	.919

*N* = 4260 couples at baseline; complete-case *N* per model in Table S1 (adjusted models retained 92.4%-92.9%; listwise deletion, no imputation). Actor = own sleep → own cognition; partner = partner’s sleep → own cognition; Time = wave (centered at baseline). *β*/*SE* from linear mixed-effects models; *p*-values from Wald *t*-tests (Satterthwaite). Adjusted for age, education, current hukou status, smoking, heavy drinking, falls, bodily pain, CES-D, retirement, multimorbidity, and social participation; wives’ models add menopausal status.

### Sensitivity Analysis

#### Standardization Approach

Actor and partner sleep coefficients were directionally and statistically consistent across the primary wave-standardized, raw-score, and baseline-referenced standardization specifications (Table S6); our conclusions do not depend on the standardization approach.

#### Expanded Covariate Set

A sensitivity analysis using an expanded covariate set (additionally including BMI, MET-min physical activity, ADL, IADL, and self-rated health) retained 48.6% of the baseline cohort across all 3 exposures and is reported in Table S7; actor and partner associations were directionally consistent with the primary specification, with statistical significance attenuated for several terms, consistent with the substantially smaller complete-case sample.

## Discussion

This is the first study examining the longitudinal dyadic effects of sleep durations on cognitive health in middle-aged and older married couples using the APIM. Three findings were most salient. First, own (actor) nocturnal sleep duration showed a robust, inverted U-shaped association with cognitive function in both wives and husbands, with an estimated optimum near 6 h per night. Second, husbands’ nocturnal sleep duration was nominally associated with wives’ cognitive function in a similar inverted U-shaped pattern, but this partner effect did not survive false discovery rate correction. Therefore, this partner effect needs to be interpreted exploratory finding warranting further investigation rather than a confirmed result. Third, post-lunch napping duration showed no significant association with cognitive function for either partner after adjustment of covariates. Overall, nocturnal sleep duration appeared to be the primary sleep-related predictor of cognitive function in older populations, with a consistent, symmetric inverted-U pattern common to both wives and husbands.

### Robust Actor Effects of Nocturnal Sleep on Global Cognition

Our analysis confirmed inverted U-shaped associations between nocturnal sleep duration and global cognition among both wives and husbands. Overall, moderate nighttime sleep plays a protective role in cognitive health regardless of demographics and health status. On the contrary, both shorter and longer sleepers had a significantly higher risk of cognitive impairment. The impact of short sleep duration on cognitive decline has been linked to the increased production and compromised clearance of dementia-related pathogenic markers, such as amyloid-β and tau protein, leading to cumulative neurodegenerative damage.^11^^,^^32^ For long sleep duration, there are some plausible explanations: First, sleep fragmentation with longer waking time at night could lead to compensatory increased sleep duration.^33^ Further, long sleep duration has been associated with elevated levels of inflammatory cytokines, such as C-reactive protein and interleukin-6, which play a crucial role in the pathogenesis of dementia.^34^ More importantly, prodromal indicators of cognitive decline, including vital exhaustion, somatic decline, and degenerative conditions such as psychiatric disorders and frailty, have been linked with long sleep duration.^8^ Evidence from a longitudinal cohort study indicates that the association between long sleep duration and incident dementia attenuated over time,^35^ which suggests that long sleep duration might serve as a marker of cognitive stress or compensatory mechanism rather than a direct risk factor. Thus, interventions should address the causes and consequences of abnormal sleep duration rather than simply encouraging long sleepers to reduce sleep time.

Methodologically, using the same CHARLS dataset either alone or combined with the English Longitudinal Study of Aging, Liu^12^ and Ma^36^ identified the inverted U-shaped associations between nocturnal sleep and risk of cognitive impairment or global cognition score. However, the age range of Liu’s study was above 60 years old, which limited their generalizations to middle-aged adults who were vulnerable to mild cognitive impairment. They also only used single-point data on baseline sleep duration and the risk of cognitive impairment while ignoring the dynamic nature of both sleep and cognition. In terms of statistical methods, Liu’s study used restricted cubic splines with knots, and Ma’s study distinguished categories of sleep duration rather than using quadratic terms. Building upon the well-established shape, our study intensified the inverted U-shaped association between nighttime sleep duration and cognitive function in adults aged above 45 years, for both wives and husbands.

### Exploratory Partner Effects of Husband’s Nocturnal Sleep on Wives’ Global Cognition

Husbands’ nocturnal sleep duration showed a nominally significant, inverted U-shaped association with wives’ global cognition after adjustment, while wives’ nocturnal sleep duration showed no significant association with husbands’ cognition after adjustment. However, this husband-to-wife association did not survive correction for multiple testing, and formal equality tests found no statistical evidence that the partner effect differed between wives and husbands. Therefore, we present this partner effect as an exploratory, hypothesis-generating observation rather than a confirmed finding of sex-specific spousal influence and interpret it with corresponding caution.

One speculative explanation drawn from the broader literature on collectivism and spousal dependency proposes that wives may depend on husbands across financial, decision-making, and social domains. In cultures valuing individualist, it is typical to observe non-significant partner effects between husbands and wives: For instance, no partner effects were observed among American couples regarding social participation and cognitive function.^37^ On the contrary, Latino cultural studies typically reported that husbands’ health was associated with wives’ health but not vice versa: For instance, a study in Mexican-American couples found that husbands’ risks of depression were positively related to wives’ depressive symptoms, but the wives’ depression was unrelated to the husbands’ depressive symptoms.^38^ Another Mexican study reported that wives’ social participation was associated with husbands’ cognitive function but not vice versa.^39^ In cultures valuing collectivism and traditional Confucianism, it is speculated that wives may exhibit a higher level of dependency on their husbands in various aspects of their lives, encompassing financial support, decision-making abilities, social roles and responsibilities, as well as expansion to cognitive activities.^31^ However, a recent review synthesizing longitudinal dyadic analysis revealed that most studies in couple dyads were conducted in North America with individualist culture,^24^ while whether actor-partner dynamics operate similarly across collectivist versus individualist cultures remains largely untested.

A more mechanistically specific possibility is that wives may engage in vigilance toward or informal caregiving activities related to their husbands’ sleep problems, which are more prevalent in men (eg, sleep apnea) and increase in prevalence with age. This possibility gains some indirect support from our baseline data: husbands in our sample had substantially higher rates of smoking and heavy drinking than wives, both established risk factors for snoring and sleep apnea,^40^^,^^41^ providing a plausible physiological basis for husbands to have more sleep-disordered breathing for wives to be exposed to and potentially responsive to. Prior cross-cultural evidence suggests that wives in older Chinese couples may be more exposed to spousal health needs and may internalize marital stressors more strongly than husbands.^22^^,^^31^ Qualitative sociological research on sleep within couples has documented that women’s own sleep and daytime functioning are frequently shaped by responsiveness to a partner’s sleep disturbances, including monitoring a partner’s breathing or waking to address a partner’s sleep problems.^42^ Furthermore, wives in our sample also had greater IADL difficulty and higher depressive symptoms than husbands at baseline; this preexisting health burden could plausibly leave wives with less capacity to buffer its cognitive impact. Future dyadic studies incorporating validated measures of partner sleep symptoms (eg, witnessed apneas, snoring frequency) and caregiving burden would be needed to test this possibility directly.

### Limited Effects of Post-Lunch Napping on Global Cognition

Napping is common in many countries such as China and Latin America and is more prevalent in older adults than in young populations. Napping may function similarly to nocturnal sleep for cognitive function by facilitating synaptic plasticity, procedural learning processes, and memory consolidation, as compensating for inadequate nighttime sleep.^43^ However, previous evidence from cohort studies on cognitive decline has been limited and inconsistent: some studies showing that a higher risk of dementia was associated with habitual long napping than non-napping,^44–46^ while others reporting that napping at noon showed a protective effect on decreasing risk of mild cognitive impairment compared to non-napping in a Chinese cohort^47^ and a systematic review synthesizing 23 observational studies suggested a possible inverted U-shaped association between daytime napping duration and cognitive function.^43^ Contributing to the mixed results in the literature, the current finding for an association between napping duration and cognition was limited and did not remain statistically significant after adjustment or correct for multiple testing. Some interventional studies have supported the protective effects of 1 session or multiple sessions of napping on short-term cognitive function such as psychomotor function, attention, and working memory,^48–50^ but the effects on long-term cognition have not been examined. Therefore, further cohort and interventional studies on napping and cognition are needed to support the possible inverted U-shaped association between daytime napping and cognitive health.

### Implications for Future Study

The robust actor effects observed in both wives and husbands indicate that sleep duration is a factor worth further investigation in relation to cognitive aging in the context of couples. However, because our findings are observational and only this single pattern of association survived correction for multiple testing while partner effects and napping associations did not survive, the present results could not provide sufficient evidence to support incorporating sleep assessment into routine cognitive screening for couples. Therefore, we emphasize the guidance for future research: longitudinal studies with repeated, objective sleep measures to clarify temporal ordering; mechanistic studies examining candidate pathways; and intervention trials to test whether modifying sleep duration toward the observed optimum (approximately 6 h) affects cognitive performance. Furthermore, our data do not provide sufficient evidence to support couple-based sleep interventions or recommend that clinicians treating 1 partner’s sleep problems routinely assess the cognitive status of the other. Future studies with adequately powered, purpose-built designs are needed to detect partner effects and determine whether spousal sleep meaningfully affects a partner’s cognitive trajectory before this could inform clinical recommendations.

### Limitations

Several limitations of this analysis should be considered. First, with respect to sample representation, most participants had agricultural hukou, lower educational level, and were not retired and were still working. This composition may limit generalizability to the broader population. In our sample, couples contribute data until they first fail to meet the household-structure criteria. Given the substantial decline in couples contributing to later waves, attribution may be related to unmeasured factors associated with both sleep and cognition trajectory, and our complete-case adjusted models may be subject to related selection bias not fully addressed by our sensitivity analyses. Second, unmeasured confounding cannot be entirely ruled out. For example, CHARLS does not include a clinical diagnosis of sleep disorders (eg, insomnia or obstructive sleep apnea), so we were unable to adjust for these conditions despite their well-documented sex differences in prevalence and effects on cognitive functioning. Third, in terms of measures and timing, sleep durations were assessed via self-report rather than objective measurements such as actigraphy or polysomnography. Additionally, cognitive function was assessed using a composite score derived from multiple tests rather than clinical diagnosis of mild cognitive impairment or dementia. While this continuous measure provides greater statistical power and sensitivity to detect subtle changes in cognition, it does not capture clinically meaningful thresholds. Also, we were unable to account for the time of day for administering the cognitive assessment, which may contribute to within-day variability, as CHARLS interview records do not consistently capture this information.

## Conclusion

Our study provides the first evidence of robust actor effects and an exploratory husband-to-wife partner effect in the sleep-cognition relationship among Chinese couples. Both wives and husbands showed a robust, inverted U-shaped association between their own sleep duration and their own cognitive function, robust to covariate adjustment and multiple sensitivity analyses. But a nominally significant association between husbands’ sleep duration and wives’ cognitive function did not survive formal testing for sex-differential effects or correction for multiple comparisons, and we interpret this as a hypothesis for future research rather than a confirmed asymmetry. These findings support own nocturnal sleep duration as a factor worth incorporating into future research on cognitive aging, and highlight dyadic sleep-cognition associations, particularly partner effects, as a direction warranting further investigation with adequately powered, purpose-built study designs before informing clinical practice.

## Supplementary Material

glag235_Supplementary_Data

## Data Availability

Public datasets from CHALRS are accessible through application. All materials (eg, coding scripts) used in this study could be shared upon request to the corresponding author.
