# Supplementary material for: Wife-husband differences in the associations between sleep durations and global cognition among middle-aged and older married couples in China: a longitudinal dyadic analysis
Source: J Gerontol A Biol Sci Med Sci. 2026 Sep 17;81(11):glag235. doi: 10.1093/gerona/glag235 (PMC13658276; doi:10.1093/gerona/glag235)
Supplement: Supplementary file 1 [file glag235_supplementary_data.docx]

**Wife-Husband Differences in the Associations Between Sleep Durations and Global Cognition Among Middle-Aged and Older Married Couples in China: A Longitudinal Dyadic Analysis**

**Table S1. Complete-Case Sample Size Used by Each Model**

| **Exposure** | **Spouse** | **Model** | **Wave-rows (N)** | **Couples (N)** | **% of baseline** |
| --- | --- | --- | --- | --- | --- |
| Nocturnal sleep | Wife | Unadjusted | 9,430 | 4,260 | 100.0% |
| Nocturnal sleep | Wife | Adjusted (primary) | 8,876 | 3,938 | 92.4% |
| Nocturnal sleep | Husband | Unadjusted | 10,133 | 4,260 | 100.0% |
| Nocturnal sleep | Husband | Adjusted (primary) | 9,547 | 3,958 | 92.9% |
| Post-lunch napping | Wife | Unadjusted | 9,431 | 4,256 | 99.9% |
| Post-lunch napping | Wife | Adjusted (primary) | 8,877 | 3,935 | 92.4% |
| Post-lunch napping | Husband | Unadjusted | 10,154 | 4,255 | 99.9% |
| Post-lunch napping | Husband | Adjusted (primary) | 9,566 | 3,954 | 92.8% |
| Nocturnal sleep | Wife | Adjusted (sensitivity) | 5,413 | 2,071 | 48.6% |
| Nocturnal sleep | Husband | Adjusted (sensitivity) | 5,909 | 2,071 | 48.6% |
| Post-lunch napping | Wife | Adjusted (sensitivity) | 5,414 | 2,070 | 48.6% |
| Post-lunch napping | Husband | Adjusted (sensitivity) | 5,932 | 2,069 | 48.6% |

Notes. Only baseline sleep duration and cognition (both partners) required complete for cohort entry (N=4,260). “Adjusted (sensitivity)” additionally adjusts for BMI, MET-minutes, ADL, IADL, self-rated health, retaining 48.6%.

**Table S2. Random-Effects Variance Components and Model Fit Statistics (Primary Adjusted Models)**

| **Exposure** | **Spouse** | **Intercept variance** | **Time-slope variance** | **Intercept-slope r** | **Residual variance** | **AIC** | **BIC** | **Log-likelihood** |
| --- | --- | --- | --- | --- | --- | --- | --- | --- |
| Nocturnal sleep | Wife | 0.2704 | 0.0046 | -0.2150 | 0.3658 | 20546.7 | 20809.1 | -10236.3 |
| Nocturnal sleep | Husband | 0.2672 | 0.0056 | -0.1675 | 0.4696 | 23876.6 | 24134.5 | -11902.3 |
| Post-lunch napping | Wife | 0.2722 | 0.0043 | -0.1845 | 0.3682 | 20685.4 | 20947.8 | -10305.7 |
| Post-lunch napping | Husband | 0.2700 | 0.0054 | -0.1229 | 0.4703 | 24057.9 | 24315.9 | -11992.9 |

Notes. AIC/BIC = Akaike/Bayesian Information Criterion (lower = better fit, within the same outcome). Random-intercept, random-slope (time) models, couple_id as clustering unit; intercept-slope r = correlation between each couple’s random intercept and slope. Fit statistics not comparable across exposures (different outcomes/constructions); reported for completeness.

**Table S3. False Discovery Rate (Benjamini-Hochberg) Adjusted p-Values, All Actor, Partner, and Time-Interaction Tests**

| **Exposure** | **Partner** | **Parameter** | **Nominal p value** | **FDR q value** |
| --- | --- | --- | --- | --- |
| Nocturnal sleep | Wife | Actor - linear | 0.687 | 0.870 |
| Nocturnal sleep | Wife | Actor - quadratic | <0.001 | **0.002** |
| Nocturnal sleep | Wife | Partner - linear | 0.864 | 0.937 |
| Nocturnal sleep | Wife | Partner - quadratic | 0.025 | 0.239 |
| Nocturnal sleep | Wife | Actor × Time - linear | 0.917 | 0.937 |
| Nocturnal sleep | Wife | Actor × Time - quadratic | 0.116 | 0.395 |
| Nocturnal sleep | Wife | Partner × Time - linear | 0.405 | 0.648 |
| Nocturnal sleep | Wife | Partner × Time - quadratic | 0.034 | 0.239 |
| Nocturnal sleep | Husband | Actor - linear | 0.045 | 0.239 |
| Nocturnal sleep | Husband | Actor - quadratic | <0.001 | **<0.001** |
| Nocturnal sleep | Husband | Partner - linear | 0.483 | 0.703 |
| Nocturnal sleep | Husband | Partner - quadratic | 0.618 | 0.847 |
| Nocturnal sleep | Husband | Actor × Time - linear | 0.593 | 0.837 |
| Nocturnal sleep | Husband | Actor × Time - quadratic | 0.333 | 0.603 |
| Nocturnal sleep | Husband | Partner × Time - linear | 0.339 | 0.603 |
| Nocturnal sleep | Husband | Partner × Time - quadratic | 0.937 | 0.937 |
| Post-lunch napping | Wife | Actor - linear | 0.122 | 0.395 |
| Post-lunch napping | Wife | Actor - quadratic | 0.086 | 0.376 |
| Post-lunch napping | Wife | Partner - linear | 0.054 | 0.258 |
| Post-lunch napping | Wife | Partner - quadratic | 0.153 | 0.432 |
| Post-lunch napping | Wife | Actor × Time - linear | 0.915 | 0.937 |
| Post-lunch napping | Wife | Actor × Time - quadratic | 0.756 | 0.907 |
| Post-lunch napping | Wife | Partner × Time - linear | 0.282 | 0.601 |
| Post-lunch napping | Wife | Partner × Time - quadratic | 0.826 | 0.937 |
| Post-lunch napping | Husband | Actor - linear | 0.112 | 0.395 |
| Post-lunch napping | Husband | Actor - quadratic | 0.198 | 0.475 |
| Post-lunch napping | Husband | Partner - linear | 0.873 | 0.937 |
| Post-lunch napping | Husband | Partner - quadratic | 0.445 | 0.686 |
| Post-lunch napping | Husband | Actor × Time - linear | 0.023 | 0.239 |
| Post-lunch napping | Husband | Actor × Time - quadratic | 0.300 | 0.601 |
| Post-lunch napping | Husband | Partner × Time - linear | 0.335 | 0.603 |
| Post-lunch napping | Husband | Partner × Time - quadratic | 0.919 | 0.937 |

Notes. FDR q value = Benjamini-Hochberg-adjusted p-value, applied jointly across all 32 tests (actor/partner linear/quadratic and time-interactions, all exposures, both spouses).

**Table S4. Predicted Global Cognition Score by Representative Nocturnal Sleep Duration (Adjusted Model)**

| **Spouse** | **Sleep hours** | **Predicted cognition (z)** | **95% CI Lower** | **95% CI Upper** |
| --- | --- | --- | --- | --- |
| Wife | 4 | 0.3149 | -0.0027 | 0.6324 |
| Wife | 5 | 0.3444 | 0.0286 | 0.6602 |
| Wife | 6 | 0.3550 | 0.0401 | 0.6699 |
| Wife | 7 | 0.3466 | 0.0321 | 0.6610 |
| Wife | 8 | 0.3191 | 0.0044 | 0.6338 |
| Wife | 9 | 0.2727 | -0.0443 | 0.5897 |
| Wife | 10 | 0.2072 | -0.1161 | 0.5305 |
| Husband | 4 | 0.6220 | 0.2993 | 0.9447 |
| Husband | 5 | 0.6528 | 0.3326 | 0.9730 |
| Husband | 6 | 0.6607 | 0.3414 | 0.9800 |
| Husband | 7 | 0.6456 | 0.3264 | 0.9648 |
| Husband | 8 | 0.6077 | 0.2876 | 0.9277 |
| Husband | 9 | 0.5467 | 0.2238 | 0.8697 |
| Husband | 10 | 0.4629 | 0.1330 | 0.7928 |

Notes. Predicted values and 95% CI from the adjusted quadratic actor-effect model; Confidence interval (CI) was calculated via delta method. Corresponds to Figure 1.

**Table S5. Sensitivity Analysis Using Restricted Cubic Spline (RCS): Nocturnal Sleep Duration and Cognition (Adjusted Model)**

**S5a. Knot Placement and Nonlinearity Test (RCS vs. Linear Actor Term)**

| **Spouse** | **N knots** | **Interior knots (h)** | **Boundary knots (h)** | **Spline df** | **χ²** | **p value** | **N couples** |
| --- | --- | --- | --- | --- | --- | --- | --- |
| Wife | 5 | 5; 6; 7 | 3; 9 | 3 | 18.133 | <0.001 | 3,938 |
| Husband | 5 | 6; 6.5; 8 | 3; 9 | 3 | 22.472 | <0.001 | 3,958 |

Notes. Restricted cubic splines fit within the primary mixed-effects model, replacing the actor’s own sleep term (partner term and covariates unchanged from the adjusted model in Table 2); knots placed at Harrell’s standard percentiles for 5 knots. Spline df, χ², and p value are from a likelihood-ratio test comparing the RCS model against the same model with the actor term forced linear (H0: linear is adequate).

**S5b. Predicted Cognition by Nocturnal Sleep Duration: RCS vs. Quadratic Model**

| **Spouse** | **Sleep hours** | **Predicted cognition (z) — RCS** | **95% CI Lower** | **95% CI Upper** | **Predicted cognition (z) — quadratic** |
| --- | --- | --- | --- | --- | --- |
| Wife | 4 | 0.3278 | 0.0085 | 0.6471 | 0.3149 |
| Wife | 5 | 0.3596 | 0.0424 | 0.6767 | 0.3444 |
| Wife | 6 | 0.3445 | 0.0289 | 0.6601 | 0.3550 |
| Wife | 7 | 0.3526 | 0.0379 | 0.6672 | 0.3466 |
| Wife | 8 | 0.3258 | 0.0106 | 0.6410 | 0.3191 |
| Wife | 9 | 0.2680 | -0.0498 | 0.5858 | 0.2727 |
| Wife | 10 | 0.2051 | -0.1204 | 0.5306 | 0.2072 |
| Husband | 4 | 0.6207 | 0.2972 | 0.9442 | 0.6220 |
| Husband | 5 | 0.6710 | 0.3497 | 0.9924 | 0.6528 |
| Husband | 6 | 0.6732 | 0.3536 | 0.9928 | 0.6607 |
| Husband | 7 | 0.6366 | 0.3167 | 0.9564 | 0.6456 |
| Husband | 8 | 0.6024 | 0.2816 | 0.9232 | 0.6077 |
| Husband | 9 | 0.5421 | 0.2184 | 0.8659 | 0.5467 |
| Husband | 10 | 0.4750 | 0.1424 | 0.8076 | 0.4629 |

Notes. RCS vs. quadratic predicted global cognition at representative nocturnal sleep durations, from the primary adjusted model; RCS 95% CI via the delta method. Both specifications place the predicted peak at 6 hours for both spouses (Figure 1), and RCS and quadratic predicted values are nearly identical in both peak location and overall magnitude (peak-to-trough range ratio ≈ 1.0 for both spouses).

**Table S6. Sensitivity Analysis: Nocturnal Sleep-Cognition Associations Under Alternative Cognition Standardizations**

| **Spouse** | **Parameter** | **Specification** | **β** | **SE** | **p value** |
| --- | --- | --- | --- | --- | --- |
| **Wife** | Actor (own sleep) - linear | Primary (wave-standardized) | -0.0026 | 0.0065 | 0.687 |
|  |  | Raw score | -0.0167 | 0.0382 | 0.663 |
|  |  | Baseline-referenced z | -0.0028 | 0.0064 | 0.663 |
| **Wife** | Actor (own sleep) - quadratic | Primary (wave-standardized) | -0.0095 | 0.0024 | <0.001 |
|  |  | Raw score | -0.0590 | 0.0141 | <0.001 |
|  |  | Baseline-referenced z | -0.0099 | 0.0024 | <0.001 |
| **Wife** | Partner (spouse’s sleep) - linear | Primary (wave-standardized) | -0.0012 | 0.0068 | 0.864 |
|  |  | Raw score | -0.0176 | 0.0399 | 0.659 |
|  |  | Baseline-referenced z | -0.0029 | 0.0067 | 0.659 |
| **Wife** | Partner (spouse’s sleep) - quadratic | Primary (wave-standardized) | -0.0053 | 0.0024 | 0.025 |
|  |  | Raw score | -0.0351 | 0.0139 | 0.012 |
|  |  | Baseline-referenced z | -0.0059 | 0.0023 | 0.012 |
| **Husband** | Actor (own sleep) - linear | Primary (wave-standardized) | -0.0146 | 0.0073 | 0.045 |
|  |  | Raw score | -0.0776 | 0.0373 | 0.037 |
|  |  | Baseline-referenced z | -0.0148 | 0.0071 | 0.037 |
| **Husband** | Actor (own sleep) - quadratic | Primary (wave-standardized) | -0.0115 | 0.0026 | <0.001 |
|  |  | Raw score | -0.0628 | 0.0131 | <0.001 |
|  |  | Baseline-referenced z | -0.0119 | 0.0025 | <0.001 |
| **Husband** | Partner (spouse’s sleep) - linear | Primary (wave-standardized) | 0.0048 | 0.0069 | 0.483 |
|  |  | Raw score | 0.0204 | 0.0352 | 0.561 |
|  |  | Baseline-referenced z | 0.0039 | 0.0067 | 0.561 |
| **Husband** | Partner (spouse’s sleep) - quadratic | Primary (wave-standardized) | -0.0013 | 0.0025 | 0.618 |
|  |  | Raw score | -0.0095 | 0.0128 | 0.462 |
|  |  | Baseline-referenced z | -0.0018 | 0.0024 | 0.462 |

Notes. N = 4,260 couples; harmonized covariate set (Table 2). Primary = wave-standardized cognition (mean 0, SD 1/wave); Raw = unstandardized score (0-31); Baseline-referenced z = standardized to Wave 1 mean/SD. Actor/partner associations directionally and statistically consistent across specifications.

**Table S7. Sensitivity Analysis: Actor and Partner Sleep-Cognition Associations Under an Expanded Covariate Set**

| **Exposure** | **Spouse** | **Parameter** | **Specification** | **β** | **SE** | **p value** | **N (couples)** |
| --- | --- | --- | --- | --- | --- | --- | --- |
| **Nocturnal sleep** | Wife | Actor - linear | Primary (covars_full) | -0.0026 | 0.0065 | 0.687 | 3,938 |
|  |  |  | Sensitivity (full covariate set) | -0.0052 | 0.0085 | 0.542 | 2,071 |
| **Nocturnal sleep** | Wife | Actor - quadratic | Primary (covars_full) | -0.0095 | 0.0024 | <0.001 | 3,938 |
|  |  |  | Sensitivity (full covariate set) | -0.0071 | 0.0031 | 0.024 | 2,071 |
| **Nocturnal sleep** | Wife | Partner - linear | Primary (covars_full) | -0.0012 | 0.0068 | 0.864 | 3,938 |
|  |  |  | Sensitivity (full covariate set) | -0.0023 | 0.0089 | 0.798 | 2,071 |
| **Nocturnal sleep** | Wife | Partner - quadratic | Primary (covars_full) | -0.0053 | 0.0024 | 0.025 | 3,938 |
|  |  |  | Sensitivity (full covariate set) | -0.0038 | 0.0032 | 0.226 | 2,071 |
| **Nocturnal sleep** | Husband | Actor - linear | Primary (covars_full) | -0.0146 | 0.0073 | 0.045 | 3,958 |
|  |  |  | Sensitivity (full covariate set) | -0.0069 | 0.0096 | 0.467 | 2,071 |
| **Nocturnal sleep** | Husband | Actor - quadratic | Primary (covars_full) | -0.0115 | 0.0026 | <0.001 | 3,958 |
|  |  |  | Sensitivity (full covariate set) | -0.0077 | 0.0035 | 0.027 | 2,071 |
| **Nocturnal sleep** | Husband | Partner - linear | Primary (covars_full) | 0.0048 | 0.0069 | 0.483 | 3,958 |
|  |  |  | Sensitivity (full covariate set) | -0.0040 | 0.0090 | 0.655 | 2,071 |
| **Nocturnal sleep** | Husband | Partner - quadratic | Primary (covars_full) | -0.0013 | 0.0025 | 0.618 | 3,958 |
|  |  |  | Sensitivity (full covariate set) | -0.0014 | 0.0033 | 0.666 | 2,071 |
| **Post-lunch napping** | Wife | Actor - linear | Primary (covars_full) | 0.0007 | 0.0004 | 0.122 | 3,935 |
|  |  |  | Sensitivity (full covariate set) | 0.0004 | 0.0006 | 0.448 | 2,070 |
| **Post-lunch napping** | Wife | Actor - quadratic | Primary (covars_full) | -1.05e-5 | 6.11e-6 | 0.086 | 3,935 |
|  |  |  | Sensitivity (full covariate set) | -4.94e-6 | 8.04e-6 | 0.538 | 2,070 |
| **Post-lunch napping** | Wife | Partner - linear | Primary (covars_full) | -0.0008 | 0.0004 | 0.054 | 3,935 |
|  |  |  | Sensitivity (full covariate set) | -0.0008 | 0.0005 | 0.131 | 2,070 |
| **Post-lunch napping** | Wife | Partner - quadratic | Primary (covars_full) | 7.58e-6 | 5.31e-6 | 0.153 | 3,935 |
|  |  |  | Sensitivity (full covariate set) | 1.20e-5 | 6.84e-6 | 0.080 | 2,070 |
| **Post-lunch napping** | Husband | Actor - linear | Primary (covars_full) | 0.0007 | 0.0004 | 0.112 | 3,954 |
|  |  |  | Sensitivity (full covariate set) | -0.0001 | 0.0006 | 0.856 | 2,069 |
| **Post-lunch napping** | Husband | Actor - quadratic | Primary (covars_full) | -7.25e-6 | 5.63e-6 | 0.198 | 3,954 |
|  |  |  | Sensitivity (full covariate set) | 7.36e-6 | 7.24e-6 | 0.310 | 2,069 |
| **Post-lunch napping** | Husband | Partner - linear | Primary (covars_full) | 0.0001 | 0.0005 | 0.873 | 3,954 |
|  |  |  | Sensitivity (full covariate set) | 0.0001 | 0.0006 | 0.926 | 2,069 |
| **Post-lunch napping** | Husband | Partner - quadratic | Primary (covars_full) | -4.92e-6 | 6.44e-6 | 0.445 | 3,954 |
|  |  |  | Sensitivity (full covariate set) | -1.95e-6 | 8.45e-6 | 0.818 | 2,069 |

Notes. Sensitivity = additionally adjusts for BMI, MET-minutes, ADL, IADL, self-rated health; N = complete-case sample per model. Associations directionally consistent between specifications; significance attenuates under sensitivity, consistent with its smaller N (48.6% vs. 92.4-92.9%).
